# Decade-long impact factors in ophthalmology journals and the effect of journal characteristics: a longitudinal study

**DOI:** 10.5935/0004-2749.20220090

**Published:** 2025-08-21

**Authors:** Sushma Nandyala, Ashik Mohamed, Sunita Chaurasia, Raja Narayanan

**Affiliations:** 1 Ophthalmic Biophysics, L V Prasad Eye Institute, Hyderabad, Telangana, India; 2 The Cornea Institute, L V Prasad Eye Institute, Hyderabad, Telangana, India; 3 Smt. Kanuri Santhamma Centre for Vitreo-Retinal Diseases, L V Prasad Eye Institute, Hyderabad, Telangana, India

The quality of published literature is evaluated by various metrics, wherein an important one is an impact factor (IF) available in Journal Citation Report (JCR). The IF range varies across fields of science^(1)^. Bibliometric trends and citation analysis in ophthalmology have been reported for 1997-2013^(2-4)^. Vainer et al. attributed an increase in IFs of ophthalmology journals to an annual increase in the number of ophthalmology journals^(1)^. Since the explosion of open access journals, there is a lack of literature on the recent trends in IFs for ophthalmology journals. Herein, we evaluated and reported the trends in IFs of ophthalmology journals over the last decade.

The data on IFs were collected from the JCRs released annually by Thomson Reuters (2009-2015) and Clarivate Analytics (2016-2020). Other information related to journals, such as geographical origin, age, publication frequency, type of content (general/special), and nature of access (open access/not), was collected from the journals’ online webpages. The geographical regions of origin were grouped under America, Europe, and Australasia. America included Brazil, Canada, and United States; Europe included Austria, Denmark, England, France, Germany, Netherlands, and Switzerland; and Australasia included Australia, mainland China, India, Iran, and Japan.

Sixty-seven ophthalmology journals had IF in the 11- year period (2009-2019). The number ranged from 46 (2009) to 60 (2019) with a median of 59 (interquartile range (IQR), 57-60), and there was no statistically significant increase during this period (p=0.07), whereas the median (1.749-1.976, 0.048/year, p=0.004, R^2^=0.63), minimum (0.510-0.605, 0.051/year, p=0.02, R^2^=0.50), and maximum (7.755-14.860, 0.469/year, p=0.003, R^2^=0.65) IFs increased significantly. Mean IF was 1.716, 1.747, 1.791, 1.733, 1.960, 1.997, 2.002, 2.208, 2.325, 2.394, and 2.510 for the years 2009-2019, respectively. The number showed a significant increase from 41 in 2000 to 58 in 2013, and the maximum IF significantly increased from 4.682 to 9.897^(1)^.

Approximately 70% had a publication scope in the areas of general ophthalmology and optometry, whereas ~30% were specialty journals focusing on specific topics, such as cataract, contact lens, cornea, eye movement, glaucoma, neuro-ophthalmology, ocular surface, ophthalmic plastic surgery, paediatric ophthalmology, refractive surgery, retina, vision electrophysiology, and visual neuroscience. The median age was 38 years (IQR, 25-166 years). American journals constituted 56.7%, whereas European and Australasian journals comprised 32.8% and 10.5%, respectively. Approximately 14.9% were open access journals. Median publication frequency was six issues/year (IQR, 6-12 issues/year). There was no effect of age (p=0.66), geographical origin (p=0.16), publication frequency (p=0.28), content type (p=0.49), and access nature (p=0.07) on longitudinal trends in IFs.

For the IF analyses as a function of time (year), journals that had at least IFs for a minimum of ten years (n=52) were considered, and their names were deidentified. Among these, 27 journals (51.9%) showed statistically significant increasing trends in IF (Table 1), whereas 24 (46.2%) showed no trend. Only one journal had a significant decrease in IF. Comparing journals with an increasing trend in IF and those without trend, there were no significant differences in journal age, geographical origin, publication frequency, content type, and access nature (Table 2). It may be a general nonspecific trend not limited to ophthalmology and can be explored in other fields.

**Table 1:** Ophthalmology journals by trends in impact factors in 2009-2019

Serial number	Journal ID	P-value	Correlation coefficient	Trend	Impact factor
1	1	**0.0007**	0.86	Increase	2.409 + 0.093^*^year
2	2	0.21		No trend	4.186 ± 0.440
3	5	**0.0003**	0.90	Increase	0.239 + 0.057^*^year
4	6	0.15		No trend	1.335 ± 0.252
5	7	**0.003**	0.80	Increase	2.700 + 0.091^*^year
6	8	0.90		No trend	1.361 ± 0.111
7	9	**<0.0001**	0.92	Increase	1.599 + 0.167^*^year
8	10	**0.007**	0.75	Increase	1.001 + 0.060^*^year
9	12	0.07		No trend	2.053 ± 0.263
10	13	**0.03**	0.64	Increase	1.434 + 0.058^*^year
11	14	**0.02**	0.70	Increase	2.465 + 0.043^*^year
12	15	0.08		No trend	1.058 ± 0.154
13	16	0.39		No trend	1.717 ± 0.397
14	17	**0.0002**	0.90	Increase	0.756 + 0.091^*^year
15	18	0.18		No trend	2.987 ± 0.230
16	19	**0.0002**	0.90	Increase	1.773 + 0.069^*^year
17	20	**0.049**	0.63	Increase	1.087 + 0.079^*^year
18	22	0.16		No trend	2.167 ± 0.169
19	23	0.10		No trend	0.932 ± 0.134
20	24	**<0.0001**	0.97	Increase	-0.201 + 0.167^*^year
21	26	0.79		No trend	3.491 ± 0.144
22	28	**0.0002**	0.92	Increase	3.266 + 0.315^*^year
23	29	**0.008**	0.75	Increase	1.133 + 0.067^*^year
24	30	0.09		No trend	0.495 ± 0.097
25	31	0.44		No trend	1.025 ± 0.150
26	32	0.70		No trend	2.642 ± 0.241
27	34	0.49		No trend	1.928 ± 0.274
28	35	**<0.0001**	0.95	Increase	1.087 + 0.141^*^year
29	36	**0.005**	0.78	Increase	1.375 + 0.049^*^year
30	38	**0.0005**	0.87	Increase	0.552 + 0.057^*^year
31	39	0.13		No trend	2.860 ± 0.455
32	40	**0.002**	-0.81	Decrease	3.055 - 0.096^*^year
33	41	**0.04**	0.62	Increase	0.496 + 0.026^*^year
34	42	0.12		No trend	2.203 ± 0.183
35	44	**0.001**	0.83	Increase	0.813 + 0.202^*^year
36	45	**0.004**	0.79	Increase	1.897 + 0.660^*^year
37	46	**0.001**	0.85	Increase	1.368 + 0.142^*^year
38	47	0.61		No trend	1.667 ± 0.502
39	48	0.41		No trend	1.341 ± 0.252
40	49	**<0.0001**	0.94	Increase	0.581 + 0.074^*^year
41	50	**0.005**	0.77	Increase	1.154 + 0.071^*^year
42	51	0.06		No trend	1.186 ± 0.339
43	52	0.41		No trend	0.716 ± 0.152
44	53	**0.002**	0.82	Increase	1.241 + 0.066^*^year
45	54	**<0.0001**	0.94	Increase	4.874 + 0.343^*^year
46	56	0.07		No trend	1.577 (1.458-1.895)
47	57	0.82		No trend	1.203 ± 0.195
48	58	**0.003**	0.80	Increase	8.097 + 0.469^*^year
49	59	**0.0005**	0.87	Increase	2.682 + 0.118^*^year
50	63	**0.0008**	0.86	Increase	2.722 + 0.137^*^year
51	66	0.74		No trend	2.179 ± 0.259
52	67	0.09		No trend	1.885 ± 0.466

This table shows the list of ophthalmology journals with at least 10 impact factors during the period of 2009-2019, the significance of the relationship between impact factors and year, Pearson or Spearman correlation co-efficient (if significant), mean ± standard deviation (if there was no trend), and linear equation with slope and intercept (if there was a significant trend). Bold values denote a statistically significant relationship at p<0.05.

**Table 2:** Comparison of ophthalmology journals with an increasing trend in impact factors with those showing no trend

Journal variable	Journals with increasing trend in impact factors (n=27)	Journals with no trend in impact factors (n=24)	P-value
Age of the journal (years), median (interquartile range)	39 (26-64)	42 (26-59)	0.99
Geographical region of origin	America - 44.5% Europe - 40.7% Australasia - 14.8%	America - 66.7% Europe - 29.2% Australasia - 4.1 %	0.21
Publication frequency (issues/year), median (interquartile range)	6 (6-12)	12 (6-12)	0.28
Type of content, General : Special	70.4%: 29.6%	62.5%: 37.5%	0.76
Open access, n (%)	1 (3.7%)	3 (12.5%)	0.33

This table shows the comparisons between journals with an increasing trend in impact factor and those without the trend by journal age, geographical region of origin, publication frequency, type of content, and nature of access. Comparisons were performed by Mann-Whitney test for continuous data and Chi-square test or Fisher’s exact test for categorical data. A p-value <0.05 was considered statistically significant.

Mansour et al. categorized the top 19 journals in ophthalmology and reported that subspecialty journals had lower IFs than general ophthalmology journals^(3)^, although it may not be appropriate to compare IFs based on content in different areas. Although subspecialty journals had a higher self-citation rate than general ophthalmology journals, the self-citation rate does not affect IF in ophthalmology, unlike in other fields^(5)^. Further, open access has not affected the citation of ophthalmology articles. This can be explained by a higher number of articles published in ophthalmology by Western countries who have subscribed access through either institution or library^(6)^.

Other possible factors that are not evaluated in our study include the number of articles published/year, availability of advance online publication, tweetations, publication time lag, content type, study nature, article length, and numbers of citations, references, and authors. Publication time lag typically does not affect the IFs of ophthalmology journals^(7)^. It was reported that bias in the positive and negative results exists in ophthalmic literature, and positive results were published in journals with higher IF^(8)^.

Although there are advantages in considering IF to analyze research content, there are also limitations. Different fields have varied IFs; for example, in 2019, the topmost journals in oncology and ophthalmology had IFs of 292.278 and 14.860, respectively. There is a varied disparity in IF across disciplines. Sometimes, even negative citations are counted for calculating IF, which means that both positive and negative citations were equally treated in the assessment^(3)^. Further, some journals are hybrid in nature that can carry variable proportions of open and closed access papers.

A 2-year IF is still a standard. IFs can be higher in longer citation windows. Some articles, known as sleeping beauties^(9)^, were recognized much later after publication. Analysis of 28 ophthalmologic journals revealed that IFs of 3- and 4-year citation windows were appropriate to analyze research quality^(10)^.

Impact factors can seriously affect editorial policies, such as accepting a greater number of review articles, coercive citation, i.e., editors forcing authors to add a spurious citation, and omission of relatively less citable items, such as a letter to editors, photo-essays, and case reports. One of the current top 15 ophthalmology journals had the highest relative increase in IF, which correlated with a decrease in the number of articles published with time. A dramatic rise in IF of one of the current top 10 ophthalmology journals is reported, which was explained by online access, editorial policy changes, and revolutionary diagnostic and therapeutic advances in subspecialty research^(3)^.

To our knowledge, this is the first study to evaluate impact factors in ophthalmology journals over the last decade and the only study that categorized journals based on their trends and evaluated various factors influencing IFs. Future studies can evaluate other factors and trends in IF during the post-COVID era and compare them with our results to understand the growth of ophthalmic literature.

## References

[1] Vainer I, Mimouni F, Blumenthal EZ, Mimouni M. (2016). Trends in impact factors of ophthalmology
journals. Indian J Ophthalmol.

[2] Sims JL, McGhee CN. (2003). Citation analysis and journal impact factors in ophthalmology and
vision science journals. Clin Exp Ophthalmol.

[3] Mansour AM, Mollayess GE, Habib R, Arabi A, Medawar WA. (2015). Bibliometric trends in ophthalmology 1997-2009. Indian J Ophthalmol.

[4] Schulz CB, Kennedy A, Rymer BC. (2016). Trends in ophthalmology journals: a five-year bibliometric
analysis (2009-2013). Int J Ophthalmol.

[5] Mimouni M, Segal O. (2014). Self-citation rate and impact factor in
ophthalmology. Ophthalmic Res.

[6] Lansingh VC, Carter MJ. (2009). Does open access in ophthalmology affect how articles are
subsequently cited in research?. Ophthalmology.

[7] Chen H, Chen CH, Jhanji V. (2013). Publication times, impact factors, and advance online publication
in ophthalmology journals. Ophthalmology.

[8] Mimouni M, Krauthammer M, Gershoni A, Mimouni F, Nesher R. (2015). Positive results bias and impact factor in
ophthalmology. Curr Eye Res.

[9] Ohba N, Nakao K. (2010). The 101 most frequently cited articles in ophthalmology journals
from 1850 to 1949. Arch Ophthalmol.

[10] Liu XL, Gai SS, Zhang SL, Wang P. (2015). An Analysis of peer-reviewed scores and impact factors with
different citation time windows: a case study of 28 ophthalmologic
journals. PLoS One.

